# Fecal microbiota transfer to treat ulcerative colitis: Medical and legal challenges

**DOI:** 10.3389/fgstr.2023.1161610

**Published:** 2023-03-23

**Authors:** Arndt Steube, Johannes Stallhofer, Andreas Stallmach

**Affiliations:** Department of Internal Medicine IV (Gastroenterology, Hepatology, and Infectious Diseases), Jena University Hospital, Jena, Germany

**Keywords:** FMT, fecal microbiota transfer (FMT), ulcerative colitis, therapy, microbiome

## Abstract

Ulcerative colitis (UC) is one of the main forms of chronic inflammatory bowel disease; however, despite intensive efforts, its etiology remains unclear. It is generally accepted that disturbances in the gastrointestinal microbiota (“dysbiosis”) contribute to the manifestation and perpetuation of UC. To date, treatment has focused on anti-inflammatory strategies; however, their widespread application is limited by side effects and primary/secondary loss of response. Following the resounding success of fecal microbiota transfer (FMT) to treat *Clostridioides difficile* infection (CDI), numerous studies have shown that FMT is also effective and safe in UC patients. In this review, we discuss the various modifications (e.g., antibiotic preconditioning, multi-donor concept, extension/intensification of application, long-term therapy, and dietary donor conditioning) that increase the efficacy of FMT. We then describe how the continuous need for healthy donors and the associated medicolegal requirements, limit the large-scale application of FMT. We conclude that FMT will likely be viewed as a transitional technology, which will be superceded by recombinantly produced bioproducts once the therapeutically active substances have been identified.

## Introduction

Ulcerative colitis (UC) is a chronic, disabling disease with relapsing-remitting symptoms such as severe bloody diarrhea with urgency, abdominal pain, and extraintestinal manifestations. 95% of UC cases implicate the colon and rectum. The disease can affect people of any age. The prevalence of UC has doubled from 1995 to 2016 and the greatest increase (2.5-fold) was seen in individuals aged over 40 years (1–3). In the United States (U.S.) and Western Europe, the prevalence of UC was estimated to be over 200 patients per 100,000 inhabitants (4, 5). Although the etiopathogenesis of UC remains elusive, substantial progress in the understanding of UC occurrence and progression has been achieved over the past few decades, which has led to the development of effective treatment strategies.

The primary goal in the treatment of UC is long-term clinical remission. Treatment strategies aim to avoid the need for colectomy and prevent the development of inflammation-triggered colorectal carcinoma. First-line treatment options for mild to moderate UC include mesalazine preparations and steroids. Patients with chronic disease are routinely treated with immunosuppressants (e.g., thiopurines), Janus kindase inhibitors, sphingosin-1-phosphate receptor (e.g., S1PR) modulators, and biologics (e.g., anti-tumor necrosis factor [TNF], anti-interleukin [IL]-12/23, and anti-integrin antibodies) (6, 7). However, this often leads to undesirable, sometimes severe side effects (8, 9). Despite these innovative treatment concepts, the frequency of UC patient hospitalization in Germany has not decreased in recent years (10). Therefore, there is an “unmet medical need” for new, effective treatment options for patients with UC, which are associated with fewer side effects.

## FMT in UC

The use of FMT in the treatment of intestinal inflammation is a very old concept. This strategy was used as early as the 4^th^ century (Dong Jin Dynasty) in Chinese medicine. The success of FMT in treating recurrent CDI, led to this concept being applied to the treatment of patients with active UC. The rationale behind the use of FMT to treat UC was that disturbances in the gastrointestinal microbiota are causally linked to the pathogenesis of UC. Numerous studies have shown that patients with active UC have significantly reduced gut microbiome diversity (11–13).

The probable first successful example of UC treatment using FMT was a self-experiment performed by a doctor with steroid-dependent UC (14). Numerous case studies/series followed, until the results of the first randomized controlled trial (RCT) of FMT in UC were published in 2015. To date, ten RCTs on FMT in UC, involving over 400 patients, have been performed. Among these, eight have examined remission induction and three have studied the maintenance of remission. The study by Haifer et al. examined both remission induction and remission maintenance. Controlled studies show remission rates of 12%–85%, compared with 5%–50% in the placebo group (15) Two of the eight induction RCTs showed negative results (16, 17), while six showed positive results (15, 18–22). A systematic review reported a pooled rate of clinical and endoscopic remission of 27.9%, with “the number needed to treat” of 5 (95% confidence interval [CI], 4–10) (23).

## Strategies to increase the efficacy of FMT in UC

In order to increase the efficacy of FMT in UC, various modifications of the concept have been discussed (see **Table 1**).

**Table 1:** Strategies to increase the efficacy of FMT in UC.

Multi-donor concept
Increased frequency and prolonged period of application	
Antibiotic preconditioning
Dietary preconditioning
Dietary maintenance therapy

The diversity of the donor microbiota and the number of transferred taxa were shown to be associated with the success of FMT in *post-hoc* analyses (24). Therefore, recent studies have selected a multi-donor approach, whereby the microbiota of different donors are pooled prior to transfer (19, 20). At the same time, the frequency of FMT was increased and the duration of therapy extended (25) in order to achieve the desired endpoint, clinical remission. A meta-analysis showed that the cumulative transfer of larger amounts of donor microbiota (> 300 g) was associated with higher FMT efficacy (26).

Another important aspect seems to be antibiotic pretreatment. It has been shown that antibiotic pretreatment before FMT increased the patient response rates to over 50% (23, 27). The concept of antibiotic pretreatment is supported by another study. A phase 1b trial of patients with UC, examined the safety and efficacy of SER-287, an oral formulation of *Firmicutes* spores. In addition, the study assessed the effect of vancomycin preconditioning on the engraftment of SER-287 species in the recipient’s colon. A higher proportion of patients in the vancomycin/SER-287 group (17.7%) achieved clinical remission at week 8 than that in the placebo/SER-287 weekly group (13.3%). Further engraftment studies demonstrated that in the vancomycin pretreatment groups, a greater number of bacterial species were detected in the recipient stool samples collected on day 10 and all subsequent time points until 4 weeks post-dosing, compared with the placebo group (P < 0.05). More frequent administration (daily *vs*. weekly) was also associated with higher remission rates (28). Since diet is one of the major determinants of gut microbiome composition, dietary manipulation is another means of optimizing FMT outcomes (29). A very recent study by Kedia et al. has demonstrated that adherence to an anti-inflammatory diet, rich in components that improve the intestinal barrier and poor in components that cause dysbiosis, sustained UC patient remission following FMT for up to 1 year (30). The importance of interventions to sustain remission after FMT has been highlighted by Haifer et al., who showed that clinical, endoscopic, and histologic remission at week 56 could not be maintained when FMT was not continued as a permanent maintenance therapy (15). Although evidence of the effect of dietary manipulation strategies in patients with UC is limited, lessons learned from exclusive enteral nutrition or exclusion diets in patients with Crohn’s disease indicate that the right diet can successfully remodel the microbiome and reduce inflammation, as long as it is maintained (31, 32). Adhering to a specific diet may be more feasible than maintaining FMT. Furthermore, the right diet may also increase the success of FMT by creating a favorable niche for donor microbiota (33). Conversely, donor dietary habits and lifestyle may also influence the success rate of FMT; it seems that rural donors provide a healthier microbiome than urban donors (30). Although a recent re-evaluation of existing evidence could not prove the hypothesis of FMT “super-donors” in inflammatory bowel disease (IBD), it cannot be ruled out that donor effects are clinically important. Large prospective clinical trials to detect donor effects are still lacking (34). In a recent RCT in adults with active UC, dietary conditioning of the donor did not affect the outcome of FMT. Interestingly, in this three-arm study, a UC exclusion diet alone appeared to achieve higher clinical remission and mucosal healing rates than single-donor FMT with or without dietary preconditioning of the donor. However, as this trial had a very low sample size with only 17, 19, and 15 patients in each arm, these results should be interpreted with caution (17).

## Legal aspects of FMT in UC

Despite various efforts to classify FMT, there is no international consensus on whether FMT should be classified as a medicinal product or as a transplant. In 2014, the European Commission stated that FMT does not fall within the scope of EU tissue and cell legislation or any other regulatory framework. Since then, there have been intensive discussions at EU level; however, no agreed approach to the classification of FMT and FMT-based products has been reached. This has led to a situation where individual member states have made national decisions on FMT classification. In the EU, there has been a tendency to classify FMT as a medicinal product (see https://www.ema.europa.eu/en/documents/report/faecal-microbiota-transplantation-eu-horizon-scanning-report_en.pdf). Like other European countries, Germany considers FMT (from the medicolegal viewpoint) as the administration of a drug or pharmaceutical product; FMT use is therefore subject to the German Law on Pharmaceuticals (Arzneimittelgesetz, AMG §2 Abs 1 Nr. 1 and Nr. 2a). In principle, the manufacture of medicinal products is subject to approval. Exceptions are medicinal products for individual therapeutic trials, if the medicinal product is manufactured under the direct professional responsibility of a physician for the purpose of personal use on specific patients (AMG, §13, 2b). Consequently, the manufacturing physician must administer the preparations themselves; wider administration or dispatch of preparations to other colleagues is prohibited. As laid down in AMG §13, only his or her personal involvement exempts this individual from the requirement for a production permit for a given medicinal product. In accordance with the good manufacturing practice (GMP) conditions outlined in §13 AMG, clinical trials of investigational medicinal products used in FMT require manufacturing authorization under the strict supervision of competent state authorities. These requirements primarily aim to standardize the manufacturing process and the quality of the FMT preparations. Thus, in Germany, FMT outside clinical trials is only permitted in patients with recurrent CDI, after all therapeutic options, including treatment with fidaxomicin and bezlotoxumab, have been exhausted.

At present, the German Federal Institute for Drugs and Medical Devices (Bundesinstitut für Arzneimittel und Medizinprodukte, BfArM) requires adherence to the donor blood and stool testing criteria shown in **Table 2**. Further exclusion criteria that apply to donors relate to pre-existing conditions, medical treatments, travel history, social factors, and family history (see https://www.bfarm.de/SharedDocs/Risikoinformationen/Pharmakovigilanz/DE/RI/2019/RI-FMT.html). A diagnostic gap persists even after extensive screening; for instance, acute infections can arise at pathogen concentrations below the technical threshold of detectability, and even in the absence of a serologic response. This gap can be (nearly) closed with a second screening, performed 8–12 weeks later. Therefore, once the donor stool sample has been processed into a therapeutic agent after the first screening, it must be kept in “quarantine” and only used for FMT if the second screening test is negative (personal communication with the BfArM in an advisory discussion on July 9, 2019). Another limitation is the requirement to ensure CMV/EBV seroconcordance between donor and recipient. It is not possible to transfer stool from EBV/CMV sero-positive donors to sero-negative patients, which leads to a significant reduction of the patient population in clinical trials. Otherwise, a donor-patient EBV/CMV serostatus match must be performed for each FMT.

**Table 2:** Donor clinical examinations.

Examination of the donor stool		
Bacteria/Fungi	Viruses	Parasites
*Salmonella*	Norovirus GI, GII	*Entamoeba histolytica*
*Shigella*	Adenovirus	*Giardia lamblia*
*Vibrio*	Astrovirus	*Cryptosporidium* sp.
*Campylobacter*	Rotavirus	*Dientamoeba fragilis*
*Yersinia*	Enteroviruses (excluding rhinoviruses)	*Blastocystis hominis*
*Clostridioides difficile* (culture) *C. difficile* toxin B (PCR)	Aichivirus	*Cyclospora, Isospora*
*Helicobacter pylori* (stool PCR)	Sapovirus (I, II, IV, V)	*Mikrosporidia*
*Listeria monocytogenes*	SARS-CoV-2	*Ova*
Enterohemorrhagic *Escherichia coli* (EHEC/STEC)		
Enteroaggregative *E. coli* (EAEC)		
Enteropathogenic *E. coli* (EPEC)		
Enterotoxigenic *E. coli* (ETEC) It/st		
Enteroinvasive *E. coli* (EIEC)		
*Plesiomonas shigelloides*		
Multi-drug-resistant organisms (MDRO): • Carbapenem-resistant *Enterobacteriaceae* (CRE) • Extended-spectrum-β-lactamase-resistant bacteria (ESBL) • Methicillin-resistant *Staphylococcus aureus* (MRSA) • Vancomycin- and glycopeptide-resistant *Enterococci* (VRE, GRE)		
*Candida auris*		

Examination of the donor blood		
Bacteria	Viruses	Parasites
*Treponema pallidum*	Cytomegalovirus (CMV)	*Strongyloides stercoralis*
	Epstein-Barr virus (EBV)	*Trichinella* sp.
	Hepatitis virus (A, B, C, E)	*Toxoplasma gondii*
	Human immunodeficiency virus (HIV) -1, -2	
	Human T-lymphotropic virus (HTLV) -1, -2)	

Because of these requirements, the BfArM recommends that stool donations are made from so-called “stool banks” (see below) and that the transfer of “fresh stool” is avoided. This procedure significantly improves patient safety and has been disseminated at national and international consensus conferences (35). Although the complexity of donor screening may be viewed critically by certain parties, it must be clearly emphasized that these measures increase the safety of the patients by avoiding the transmission of pathogens. Independently of this, a commercially available preparation for FMT in recurrent CDI has been recently approved by the U.S. Food and Drug Administration. For this, stool derived from an intensively and repeatedly tested donor pool, has been processed into an enema, which is delivered to the patient (deep-frozen) *via* pharmacies for self-administration. However, further studies are needed to show whether this complex concept can also be implemented in patients with UC, considering the need for repeated application.

It is likely that the medicolegal assessment of FTM will change in the near future. Overall, the assessment of FMT by regulatory authorities in Europe is very heterogeneous, meaning that the availability of FMT depends on the patient’s place of residence in the EU. On July 14, 2022, the European Commission proposed new rules (within the EU legislation on blood, tissues and cells) to regulate the handling of all substances of human origin (SoHO) in the future. The aim is to enable more patients across Europe to access the treatments that they need, regardless of where they live, with a regulation that is applicable in all EU member states. In addition, the cross-border exchange of these applications, as well as the cross-border cooperation between the health authorities, should be facilitated; at the same time, a common approach ensures uniformly high quality and safety standards for all SoHO.

## Donor recruitment for FMT

**Figure 1:**
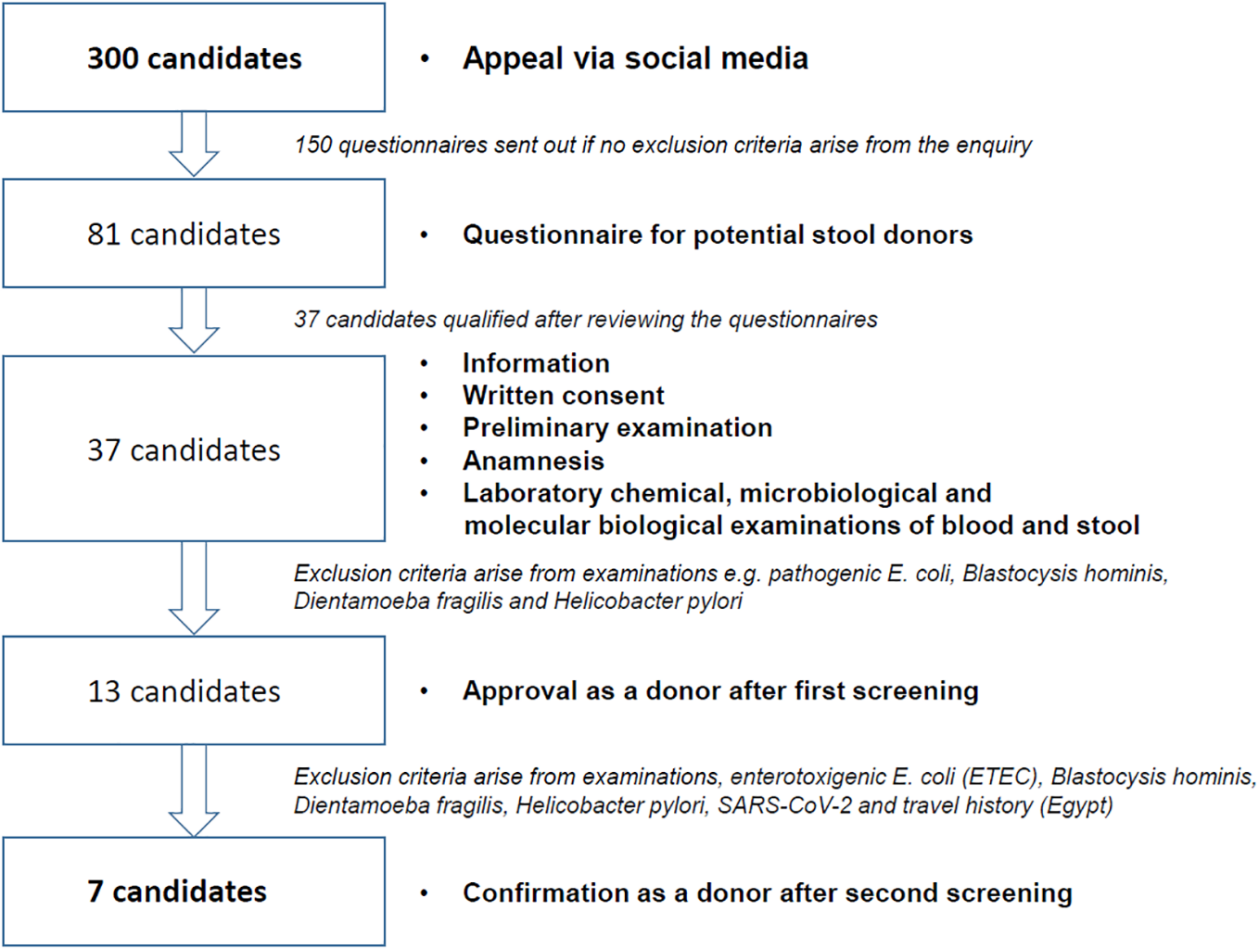
Overview of FRESCO trial donor recruitment, selection, and clinical testing.

The effort required to identify and maintain a stable donor cohort for FMT is extremely high. Our own experience in the selection of suitable stool donors shows that an immense amount of time and effort goes into the preparation of a FMT panel for the required clinical examinations of the BfArM (**Table 2**). These clinical investigations are also subject to special requirements (for validation and qualification in accordance with GMP), which may require the use of external reference laboratories. On April 1, 2020, the BfArM adapted its FMT safety requirements in view of the severe acute respiratory syndrome coronavirus 2 (SARS-CoV-2) pandemic and the potential risk of the virus being transmitted through stool donations. This means that donor stools now need to be tested for SARS-CoV-2 infection. In addition, it is challenging to identify potential donors prior to screening who are in good health and who, for example, do not have allergies or take medication. In our own experience of recruiting donors for the ongoing FRESCO trial (36), only 7 out of 300 potential candidates were suitable donors after the pre-selection and clinical testing stages (**Figure 1**)

## Solutions to current problems

To provide reliable and safe FMT therapies for patients, a strict standardization (characterization and reproducible manufacturing) and quality control for FMT medical products is required. Central biobanks or stool banks could accomplish this standardization by providing a repository of donor material that can be used in clinical trials and research studies. First stool banks have already been established. Openbiome (https://openbiome.org/), for example, a non-profit organization in the United States, is committed to providing an internationally standardized public stool bank for microbial treatments. But standardization includes both donor characterization and the subsequent processing of the stool donation into the final medical product. There is consensus that donors must be tested for all critical pathogens that can impact the safety of the treatment. Unfortunately, there is no standard for the processing of the stool donations. The procedures vary widely, from the processing of a classic stool suspension (with all components) to the enrichment of the bacterial fraction or even sterile filtration, which has been proven to contain viral and other components (37). Overall, standardization is essential for ensuring the safety and efficacy of FMT therapies and clinical studies are urgently needed to provide clarity regarding active components in order to target them. The future of more specific and limited bacterial or viral transfer is promising with the advance of biotechnology. Once the active components are identified, it will become possible to produce them biotechnologically and use them in a more targeted manner.

The use of more patient-friendly methods of administration is particularly relevant in FMT therapies. In the past, FMT was typically performed *via* colonoscopy or nasogastric tube, which can be uncomfortable and invasive for the patient. FMT-capsules are currently the preferred method of administration with obvious advantages (15). This method is non-invasive and has a high level of patient acceptance. In addition, capsules can be combined from multiple donors, which allows for a wider range of microbiota to be transferred and also offers the possibility of long-term transfer options, allowing patients to take capsules over a period of weeks or months. The number of capsules needed may vary depending on the patient’s condition and the severity of the disease. Studies are needed to establish the optimal dosage that will provide the most significant therapeutic effect while minimizing any potential adverse effects. Furthermore, whereas short-term FMT studies have shown promising results, the long-term effects of repeated FMT-capsule administration are not known sufficiently (15). In summary, clinical trials, particularly dose-finding studies, are necessary to establish the optimal dosage and duration of FMT therapies.

## Conclusion

There is no doubt that FMT is an effective and safe treatment concept for achieving remission and for preventing recurrence in at least a subgroup of patients with UC. However, the repeated and prolonged repopulation of the patient microbiome appears to be necessary for long-term UC remission. All endoscopic therapy methods are not compatible with the concept of continuous application, as they overtax patient adherence. Strategies to encapsulate the donor microbiome (e.g., lyophilization, with the option of storing the capsules in a normal household refrigerator) would enable FMT *via* the oral route and improve the practicability of the treatment method. The prerequisite for this would be the continuous availability of healthy donors who meet medicolegal requirements. However, the plasticity of the microbiome and the sensitivity of the required molecular genetic tests (e.g., the extensive donor inclusion and exclusion criteria), limit the concept considerably. The successful implementation of a large-scale stool-donor-based therapeutic strategy therefore seems questionable. Since the industrially produced FMT preparations also rely on the availability of suitable donors, they do not solve the problems associated with conventional FMT. It is therefore our task to identify the therapeutically active components of the donor microbiome in the context of FMT and to produce them recombinantly. Such “super probiotics” would provide patients with UC with alternative treatment options. It is therefore likely that conventional FMT is not here to stay.

## Author contributions

ArS, JS, and AnS contributed to conception, design and the first draft of the manuscript. All authors contributed to the article and approved the submitted version.
